# Molecular detection of porcine circovirus (PCV2 and PCV3), torque teno swine virus 1 and 2 (TTSuV1 and TTSuVk2), and histopathological findings in swine organs submitted to regular slaughter in Southeast, Brazil

**DOI:** 10.29374/2527-2179.bjvm000623

**Published:** 2023-07-26

**Authors:** Amanda Eduarda de Souza, Ana Claudia de Menezes Cruz, Ingrid Lyrio Rodrigues, Eulógio Carlos Queiroz de Carvalho, Rafael Brandão Varella, Raphael Mansur Medina, Rachel Bittencourt Ribeiro Rodrigues, Renato Luiz Silveira, Tatiana Xavier de Castro

**Affiliations:** 1 Veterinarian, Programa de Pós-Graduação em Microbiologia e Parasitologia Aplicadas (PPGMPA), Departamento de Microbiologia e Parasitologia (MIP), Universidade Federal Fluminense (UFF). Niterói, RJ. Brazil.; 2 Veterinarian, MSc. PPGMPA, MIP, UFF. Niterói, RJ. Brazil.; 3 Veterinarian, DSc. Faculdade de Veterinária, Departamento de Zootecnia (MMO), UFF. Niterói, RJ. Brazil.; 4 Veterinarian, DSc. Universidade Estadual Norte Fluminense (UENF), Campos dos Goytacazes, RJ, Brazil.; 5 Veterinarian, DSc. MIP, UFF. Niterói, RJ. Brazil.; 6 Veterinarian, MSc. Autonomus, Campos dos Goytacazes, RJ, Brazil.; 7 Veterinarian, DSc. Departamento de Morfologia (MMO), UFF. Niterói, RJ. Brazil.

**Keywords:** Histopathology, nested PCR, coinfection, pig, asymptomatic infection, Histopatologia, Nested PCR, Coinfecção, Porco, Infecção assintomática

## Abstract

Porcine circovirus 2 and 3 (PCV2 and PCV3) and torque teno sus virus 1 and 2 (TTSuV1 and TTSuVk2) are important pathogens in pig associated with post-weaning mortality, different clinical syndromes in adults (PCVAD), and a decrease of average daily weight gain (PCV2-SI) but little is known about the infection on asymptomatic pigs. The aim of this study was to evaluate the presence of PCV2, PCV3, TTSuV1, and TTSuVk2 in swine organ samples from asymptomatic pigs slaughtered in Espírito Santo State, South-eastern Brazil, through molecular detection and histopathological analysis. Nested PCR showed the presence of PCV2 DNA in 10% (14/140), PCV3 in 13.6% (19/140), TTSuV1 in 12.9% (18/140), and TTSuVk2 in 30% (42/140) of the tissue samples. All four viruses were detected in the lung, kidney, lymph node, and liver. TTSuVk2 was detecded in 30% (42/140), PCV3 in 13.6% (19/140), TTSuV1 in 12.9% (18/140), and PCV2 in 10% (14/140) of the samples. Single infections were observed in 30.7% (43/140), while co-detections in the same tissue occurred in 15.7% (22/140). The most frequent combinations were TTSuV1/TTSuVk2 in 31.8% (7/22), PCV2/TTSuVk2 in 18.1% (4/22), and PCV2/PCV3/TTSuVk2 in 13.6% (3/22). Lymphocyte depletion was associated with TTSuVk2 infection (p = 0.0041) suggesting that TTSuVK2 plays an induction of PMWS-like lymphoid lesions in pigs. The data obtained in this study show that PCV2, PCV3, TTSuV1, and TTSuVk2 are related to infection in asymptomatic animals with different tissue lesions, and the molecular diagnosis for these pathogens should be considered in the sanitary monitoring of herds.

## Resumo

O circovírus suíno 2 e 3 (PCV2 e PCV3) e os Torque Teno vírus suínos 1 e 2 (TTSuV1 e TTSuVk2) são patógenos importantes na suinocultura associados a diferentes síndromes clínicas e morte de leitões pós desmame (PCVAD) e redução no ganho diário de peso (PCV2-SI). Entretanto, pouco se sabe sobre a circulação desses agentes e o impacto da infecção em porcos assintomáticos. O objetivo deste estudo foi avaliar a presença de PCV2, PCV3, TTSuV1 e TTSuVk2 em amostras de órgãos de suínos assintomáticos abatidos no estado do Espírito Santo, região sudeste do Brasil, por meio de detecção molecular e análise histopatológica. A análise tecidual por nested PCR mostrou a presença de DNA de PCV2 em 14 (10%), PCV3 em 19 (13,6%), TTSuV1 em 18 (12,9%) e de TTSuVk2 em 42 (30%) das amostras. Todos os quatro vírus foram detectados no pulmão, rim, nódulo linfático e fígado TTSuVk2 foi detectado em 30% das amostras teciduais (42/140), PCV3 em 13.6% (19/140), TTSuV1em 12.9% (18/140), e o PCV2 em 10% (14/140. Mono infecções foram observadas em 30.7% (43/140) das amostras enquanto infecções múltiplas observadas em 15.7% (22/140 das amostras de tecido). As combinações mais frequentes foram TTSuV1/TTSuVk2 em 31.8% (7/22), PCV2/TTSuVk2 em 18.1% (4/22), e PCV2/PCV3/TTSuVk2 em 13.6% (3/22). A depleção de linfócitos foi associada à infecção por TTSuVk2 (p = 0,0041) e esses achados sugerem que TTSuKV2 desempenha uma indução de lesões linfoides semelhantes a PMWS em porcos. Os dados obtidos neste estudo mostram que PCV2, PCV3, TTSuV1 e TTSuVk2 estão relacionados à infecção em animais assintomáticos com lesões teciduais diversas, e sugerem que o diagnóstico molecular para esses patógenos deve ser considerado no monitoramento sanitário dos rebanhos.

## Introduction

Porcine circovirus type 2 (PCV2) is a major contributor to economic losses in pig farming worldwide, and is associated with postweaning multisystemic wasting syndrome (PMWS), a systemic condition that affects nursery and growing pigs in unvaccinated herds. PCV2 is also related to other clinical conditions or PCV2 associated diseases (PCVAD) affecting respiratory, enteric, and reproductive tracts, being experimentally reproduced to varying degrees (Dal Santo et al., 2020; Opriessnig et al., 2020; Segalés, 2012). The use of vaccines in PCVAD scenarios has led, on most farms, to a PCV2 subclinical infection with an absence of clear noticeable clinical signs at the moment of physical examination, none or minimal histopathological lesions in tissues, the presence of a low amount of PCV2 in tissues, and a decrease of average daily weight gain (PCV2-SI) (Oliver-Ferrando et al., 2016; Segalés, 2012).

In 2015, a new porcine circovirus, porcine circovirus type 3 (PCV3), was described in sows with porcine dermatitis and nephropathy-like syndrome (PDNS) in domestic and wild pigs in several countries, including Brazil (Cruz et al., 2020; Rodrigues et al., 2020), and associated with PMWS, respiratory and digestive signs similar to PCVAD. The detection of the virus in PCV2-SI animals also suggests the involvement of PCV3 in this syndrome (Saraiva et al., 2019; Zhai et al., 2017). Experimental infection by PCV3 suggests that this new pathogen is associated with clinical features of a PCVAD-like syndrome (Dal Santo et al., 2020; Shen et al., 2018). A recent study performed on piglets from Southern Brazil reported PCV3 associated with neonatal piglet losses (Molossi et al., 2022).

Torque teno sus virus belongs to the *Anelloviridae* family, and TTSuV1 and TTSuVk2 are related to infections in domestic swine and feral pigs (Ouyang et al., 2019). TTSuV infection of gnotobiotic pigs results in organ damage and exacerbation of coinfections, with experimental evidence of pathogenicity (Webb et al., 2020). However, the involvement of TTSuVs in PCVAD cases is a subject of debate among authors (Aramouni et al., 2010; Kekarainen & Segalés, 2012; Ramos et al., 2018).

The economic impact of PCV2 and PCV3 infection, in combination or not with TTSuV1 and TTSuVk2 in asymptomatic pigs, results in reduced weight gain and increased susceptibility to other pathogens, and increased animal maintenance costs (Alarcon et al., 2013; Wilfred et al., 2018). Minimizing the negative effects of subclinical PCV2 infection through appropriate preventive and control strategies is a challenge to veterinarians (Maes, 2012).

The use of histopathology techniques in association with PCR diagnosis has been an important source of information for sanitary monitoring of pigs to identify and monitor the circulation of these agents (Arenales et al., 2022). However, there are few data on the circulation of PCV2, PCV3, TTSuV1, and TTSuVk2 subclinical infection in animals submitted to regular slaughter. Therefore, the aim of this study was to perform molecular detection of PCV2, PCV3, TTSuV1, and TTSuVk2 in tissues from pigs submitted to regular slaughter in the South-eastern region of Brazil, correlating with the histopathological findings.

## Materials and methods

### Study design

From June 2017 to May 2018, 35 swine carcasses of approximately 150 days, from pig farms in Espírito Santo State, Southeast Brazil, were selected for this study. On in every five carcasses were selected, maintaining the slaughter line speed rate and interval between the carcasses established by the slaughterhouse. Animals were in good general condition at the pre-slaughter inspection. It was not possible to access the clinical history and vaccination protocol of these animals prior to slaughter. The study was approved by the Ethics Committee on Animal Use of the Fluminense Federal University (Protocol No. 1013/2018).

### Sample collection

Samples of lungs, kidneys, liver, and lymph nodes from each animal were collected in duplicate, totaling 280 samples, and were divided for molecular diagnosis and histopathology analysis. The choice of these organs was based on the results obtained by different authors (Opriessnig et al., 2007; Segalés, 2012; Zanella et al., 2006) and because they were considered the most relevant for investigation of PCVs and TTSuVs.

For histopathological diagnosis, the samples were placed in labeled jars containing 10% buffered formalin solution and sent to the Animal Morphology and Pathology Laboratory (LMPA- UENF) for paraffin-embedded routine staining.

### DNA extraction and molecular diagnosis of PCV2, TTSuV1, and TTSuVk2

For molecular diagnosis testing, fresh samples were stored in sterile flasks and kept under −20 ^o^C until processing. After thawing, samples were cut into smaller fragments (approximately 2 grams) and then macerated prior to tissue digestion processing. For this, the macerated tissues were placed in 2.0-mL microtubes with 150 μL of Tissue Lysis Buffer ATL (QIAamp^®^) and 50 μL of proteinase K for 1 hour at 56 ^o^C. The digested samples were subsequently submitted to DNA extraction using the Boom method (Boom et al., 1990). PCV2 nested PCR was performed by amplifying a 255-bp capsid gene fragment according to Kim and Chae (2001). Nested PCR was also performed for PCV3 by amplifying a 648 bp fragment from the capsid protein gene (ORF2) (Ku et al., 2017), followed by amplification of an internal fragment of 205 pb, as described by Cruz et al. (2020).

For TTSuV1 and TTSuVk2, a partial sequence of the non-coding region was amplified, resulting in amplicon products of 309 bp and 252 pb, respectively (Martínez-Guinó et al., 2009). As a positive control of the PCR reaction, samples previously sequenced and deposited in the GenBank database (KX833728-KX833813), obtained in an earlier study (Cruz et al., 2016), were used. MilliQ Water was used as a negative control.

### Statistical analysis

The collected data were stored in a Microsoft Excel 2010^®^ spreadsheet and analyzed with the SPSS 20 (SPSS, Inc., Chicago, IL) program. Fisher’s exact test was used to examine associations between categorical variables; P values < 0.05 were considered significant.

## Results

**Figure 1:**
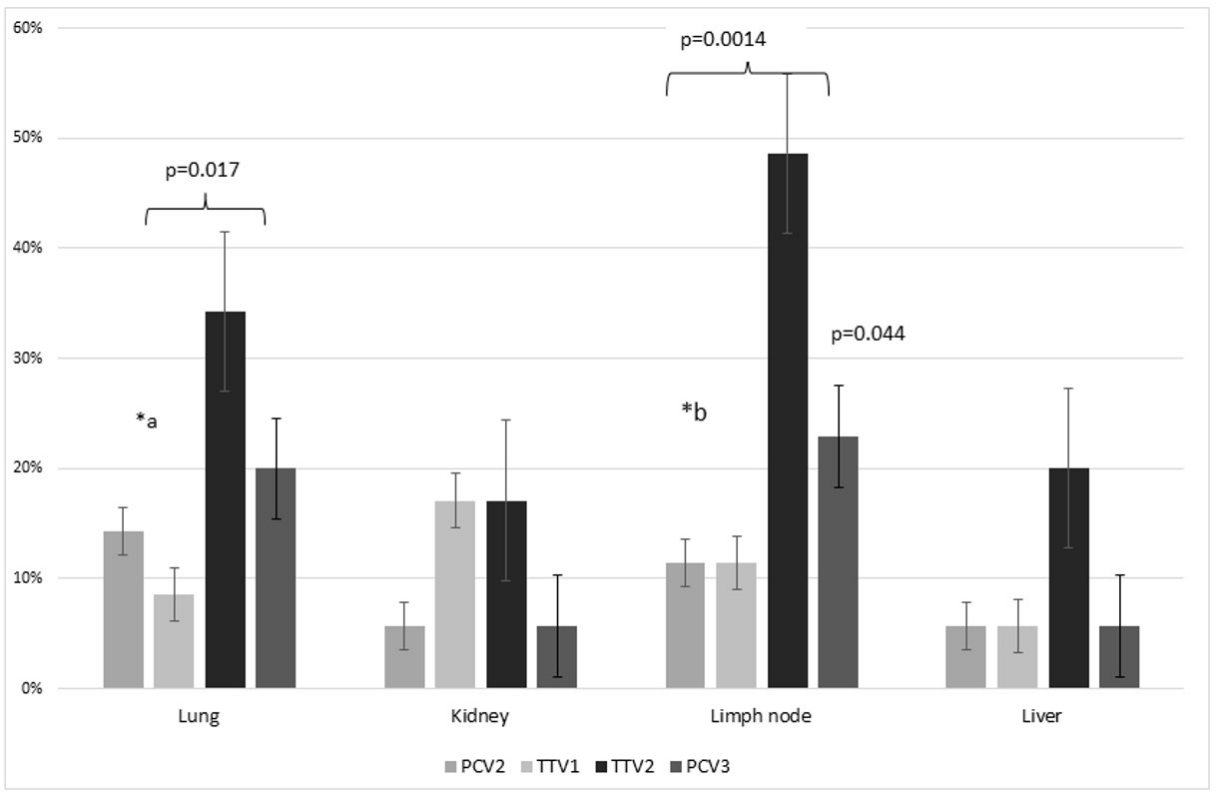
Frequency of PCV2, TTSuV1 and TTSuVk2 detection by tissue. ^*a^TTSuVk2 vs TTSuV1 (p=0.017); ^*b^TTSuVk2 vs PCV2 and TTSuV1 (p=0.0014) and PCV3 (p=0.044).

The results are presented by animals and tissues. Overall, 65.7% (23/35) of the animals were infected with one or more of the viruses included in this study. TTSuVk2 was detected in 67.7% (23/35 p = 0.0037), followed by PCV3 in 37.1% (13/35), TTSuV1 in 40% (14/35), and PCV2 in 28.6% (10/35) by the nested PCR (Table 1). The tissue analysis showed the presence of TTSuVk2 in 30% (42/140), PCV3 in 13.6% (19/140), TTSuV1 in 12.9% (18/140), and PCV2 in 10% (14/140) of the samples (Table 1). Single infections were observed in 30.7% (43/140) of samples while co-detections in the same tissue occurred in 15.7% (22/140). The most frequent combinations were TTSuV1/TTSuVk2 in 31.8% (7/22), PCV2/TTSuVk2 in 18.1% (4/22), and PCV2/PCV3/TTSuVk2 in 13.6% (3). A viral survey by tissue showed that all four viruses were detected in lung, kidney, lymph node and liver (Figure 1). Apart from TTSuV1, which was equal to TTSuVk2 detection in kidney, TTSuVk2 was the most frequent viral agent per tissue, being statistically more frequent in lung than TTSuV1 (p = 0.017) and in lymph node than PCV2 and TTSuV1 (p = 0.0014) and PCV3 (p = 0.044). TTSuVk2 was more frequent than PCV2 (p = 0.0037) and PCV3 (p = 0.030) by animal and more frequent than PCV2 (p = 0.0001), PCV3 (p = 0.0013), and TTSuV1 (p = 0.0007) by tissue.

**Table 1:** Frequency of PCV2, TTSuV1 and TTSuVk2 detection by animal and tissue.

**Variable n(%)**	**PCV2**		**PCV3**		**TTSuV1**		**TTSuVk2**	
	+	-	+	-	+	-	+	-
**Animal (35)**	10 (28.6)	25 (71.4)	13 (37.1)	22 (62.9)	14 (40)	21 (60)	23^1^^,^^4^ (67.7)	12 (34.3)
**Tissue (140)**	14 (10)	126 (90)	19 (13.6)	121 (86.4)	18 (12.9)	122 (87.1)	42^2^^,^^3^^,^^5^ (30)	98 (70)

1 TTSuVk2 vs PCV2 (p=0.0037);

2 TTSuVk2 vs PCV2 (p=0.0001);

3 TTSuVk2 vs TTSuV1 (p=0.0007);

4 TTSuVk2 vs PCV3 (p=0.0306);

5 TTSuVk2 vs PCV3 (p=0.0013).

Although the presence of PCV2, PCV3, TTSuV1, and TTSuVk2 DNA in kidney and liver was not statistically different (Figure 1), PCV2 and TTSuVk2 were more frequent in the presence of renal lesions [p = 0.021(95% CI: 1.29-6.32) and p = 0.023 (95% CI: 2.25-7.53)], respectively; and hepatic lesions by TTSuVk2 [p = 0.021 (95% CI: 1.29-6.32)] (Table 2), considering the total number of lesions observed in the histopathology per tissue. No associations between co-detections involving two, three, or four viruses and tissue lesions were found (Table 2

**Table 2:** Tissue lesion by histopathological analysis according to virus detection.

**Tissue**		**PCV2**			**PCV3**			**TTSuV1**			**TTSuVk2**			Co-detection*		
	**lesion**	+	-	*p*	+	-	*p*	+	-	*p*	+	-	*p*	+	-	*p*
**Liver**	+	1	11	0.630	1	1	0.630	1	11	0.630	5	7	**0.021** 1	3	2	0.167
	-	1	22		11	22		1	22		2	21		0	5	
**Lungs**	+	4	26	0.693	5	25	0.227	2	28	0.342	9	21	0191	1	8	0.576
	-	1	4		2	3		1	4		3	2		2	6	
**Limph node**	+	4	24	0.288	7	21	0.546	3	25	0.791	15	13	0.410	7	12	0.523
	-	0	7		1	6		1	6		2	5		0	3	
**Kidney**	+	2	25	**0.021** 2	0	10	0.357	2	23	0.799	4	23	**0.023** 3	1	6	0.559
	-	0	8		2	23		6	6		2	6		3	4	

1 Relative Risk: 2.85 (95% CI: 1.29-6.32);

2 Relative Risk: 4.12 (95% CI: 2.25-7.53);

3 Relative Risk: 3.22 (95% CI: 1.29-8.00);

* Co-detection involving two or more viruses.

The histopathological findings in the tissue samples from carcasses are summarized in Table 3. It was not possible to attribute a determined type of lesion to any specific virus, probably due to the variety of alterations observed. Nonetheless, lymphocyte depletion was associated with TTSuVk2 infection (p = 0.0041).

**Table 3:** Histopathological findings per tissue.

**Tissue**	**Histopathological findings**	**(% and positive/total)**
**Liver (n=35)**	Ductal hyperplasia	14.3% (5/35)
	Hydropic degeneration	8.6% (3/35)
	Ductal hyperplasia/ Hydropic degeneration/hepatitis	5.7% (2/35)
	Mononuclear infiltrate	5.7% (2/35)
**Lungs (n=35)**	suppurative bronchopneumonia	17.1% (6/35)
	emphysema	11.4% (4/35)
	Intersticial pneumonia	31.4% (11/35)
	Mononuclear infiltrate	20% (7/35)
	suppurative bronchopneumonia/ emphysema	5.7% (2/35)
**Limph nodes (n=35)**	lymphocyte depletion	80*% (28/35)
	inflammatory infiltrate	2.9% (1/35)
**Kidney (n=35)**	Mononuclear infiltrate	14.3% (5/35)
	Discrete intersticial nephritis	14.3% (5/35)

* p=0.0041 for TTSuVk2.

## Discussion

In this study, TTSuV infections were most frequently detected in single or coinfections. One of the explanations for these results is the high probability of detecting TTSuVs in older animals, as the prevalence of viral infection increases with time (Aramouni et al., 2010; Rogers et al., 2017; Sibila et al., 2009). A higher transmissibility, as well as a greater genetic variability in the TTSuVk2 strains, could also explain these findings, since the animal constantly experiences new TTSuV infections (Cortey et al., 2012; Ramos et al., 2018).

As in the lung, TTSuVk2 was more frequently observed in the lymph nodes than PCV3, PCV2, and TTSuV1, associated with lymphoid depletion. This association is extremely relevant, since this is the most characteristic histopathological change in the lymphoid organs of PMWS-affected pigs (Ellis et al., 1998; Rosell et al., 2000). It is speculated that, due to similar characteristics between PCV2 and TTSuVs, it also replicates in actively dividing cells by the use of host cell polymerase (Lee et al., 2012). Similar results were found by Lee and collaborators (2015), who described lymphoid depletion in PCV2-negative animals being associated with TTSuVk2, but not with TTSuV1.

These findings suggest that TTSuVk2 is involved in the induction of PMWS-like lymphoid lesions in pigs, which may explain why some pigs displayed clinical signs of wasting, but had a low or absent level of PCV2 detection in this study (Lee et al., 2012).

The four agents (PCV2, PCV3, TTSuV1, and TTSuVk2) had similar detection rates in the kidney and liver; however, TTSuVk2 was the main agent associated with injury in these two organs. Recently, a metagenomic study performed with livers collected from slaughtered pigs in Brazil revealed a high diversity of ssDNA viruses, and TTSuVs were the most commonly detected, but the presence of damage to these organs has not been investigated (Silva et al., 2020).

There are few reports on TTSuVk2 diagnostics based on molecular methods (PCR and qPCR) supporting histopathological diagnosis (Brassard et al., 2013; Lee et al., 2015). TTSuVk2 may act as a co-pathogen of PCV2 infections, influencing the development of porcine circovirus diseases (Lee et al., 2015). However, it is possible that TTSuVk2 may cause, alone or in coinfection with other viruses, lesions such as glomerulonephropathy, lymphohistiocytic infiltrates in the liver and interstitial pneumonia, respectively, with no clinical symptoms at the moment of necropsy (Aramouni et al., 2013). With regard to coinfections, the literature findings are limited to viral load data only, not supported by histopathological analysis. According to some authors, TTSuVk2 acts as a co-pathogen of PCV2 infection, influencing the development of porcine circovirus diseases (Lee et al., 2015). Our results showed no associations between coinfections involving two or three viruses and tissue injury, and no statistical value was associated with coinfection.

Our study found that an animal infected with TTSuVk2 is 2.85 times (95% CI: 1.29-6.32) more likely to have liver damage and 3.22 times (95% CI: 1.29-8.00) more likely to have kidney damage. A PCV2 infected animal is 4.12 (95% CI: 2.25-7.53) times more likely to have kidney damage. This suggests that even if the lesions are caused by these agents, they may act as facilitators of other pathogens that may or may not be related to the injuries found. Based on these results, animals infected with both TTSuV and PCV2 are more immunologically impaired and therefore more likely to become infected with secondary pathogens.

The possible limitations of our study relate to the putative presence of other important infectious agents in pig farming, such as *Mycoplasma hyopneumoniae*, a causative agent of porcine respiratory disease complex frequently reported in Southeast Brazil in combination with other bacterial or viral agents (Balestrin et al., 2022), which were not investigated. Additionally, the cross-sectional design of the investigation did not allow observation of the dynamics of infections and their associations with the lesions observed in the tissues. Furthermore, due to the potentially changing character of the presence of these viral agents in tissues, their role in single or mixed infection in tissues remains unclear and therefore, the action of other viral agents that have not been researched cannot be ruled out. Another limitation of this study was the lack of clinical history and vaccination protocol of these animals prior to slaughter, since vaccination for PCV2 is usually performed on pig farms in the region. Nonetheless, it is remarkable that some associations might be inferred between the presence of the viral agents diagnosed and microscopical lesions in clinically healthy pigs.

## Conclusions

The data obtained in this study show that PCV2, PCV3, TTSuV1, and TTSuVk2 are ubiquitous agents that circulate in clinically healthy animals and are associated with important histopathological lesions, potentially compromising performance data and records; TTSuVk2 is associated with lymphoid depletion and pulmonary lesions, similar to those of PCV2 infection.
